# ChatGPT and generative AI are revolutionizing the scientific community: A Janus‐faced conundrum

**DOI:** 10.1002/imt2.178

**Published:** 2024-02-27

**Authors:** Zhongji Pu, Chun‐Lin Shi, Che Ok Jeon, Jingyuan Fu, Shuang‐Jiang Liu, Canhui Lan, Yanlai Yao, Yong‐Xin Liu, Baolei Jia

**Affiliations:** ^1^ Xianghu Laboratory Hangzhou China; ^2^ ANGENOVO Oslo Norway; ^3^ Department of Life Science Chung‐Ang University Seoul Republic of Korea; ^4^ Department of Genetics, University Medical Center Groningen University of Groningen Groningen The Netherlands; ^5^ State Key Laboratory of Microbial Resources, Institute of Microbiology Chinese Academy of Sciences Beijing China; ^6^ State Key Laboratory of Microbial Technology Shandong University Qingdao China; ^7^ School of Life Science and Technology Wuhan Polytechnic University Wuhan China; ^8^ R‐Institute Co. Ltd. Beijing China; ^9^ Shenzhen Branch, Guangdong Laboratory of Lingnan Modern Agriculture, Genome Analysis Laboratory of the Ministry of Agriculture and Rural Affairs, Agricultural Genomics Institute at Shenzhen Chinese Academy of Agricultural Sciences Shenzhen China

## Abstract

The advent of generative artificial intelligence (AI) technologies marks a transformative moment for the scientific sphere, unlocking novel avenues to elevate scientific writing's efficiency and quality, expedite insight discovery, and enhance code development processes. Essential to leveraging these advancements is prompt engineering, a method that enhances AI interaction efficiency and quality. Despite its benefits, effective application requires blending researchers' expertise with AI, avoiding overreliance. A balanced strategy of integrating AI with independent critical thinking ensures the advancement and quality of scientific research, leveraging innovation while maintaining research integrity.

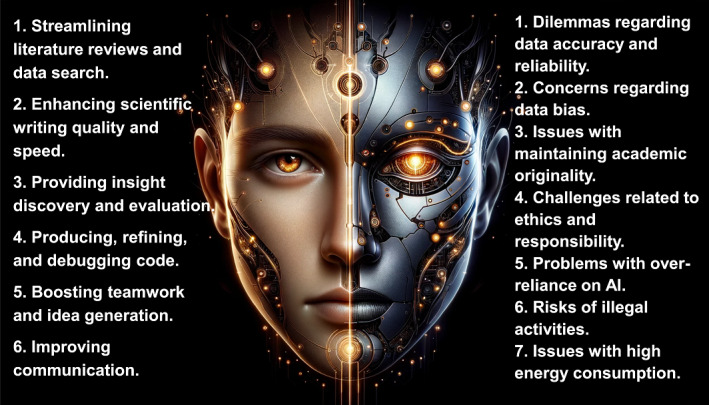

Since the launch of chatbot ChatGPT by OpenAI (November 2022), generative artificial intelligence (AI) using large language models (LLMs) has increasingly penetrated the scientific community. A survey reported in February 2023 revealed that 80% of respondents use AI chatbots [1]. By October 2023, about 30% of postdocs were employing AI chatbots for tasks like text refinement, code generation, editing, and literature management in their fields [2]. By the end of 2023, “Nature” journal recognized ChatGPT as one of the 10 pivotal contributors to science that year. In biomedical research, generative AI is revolutionizing drug discovery and development by predicting molecular structures and biological interactions [3]. It is also instrumental in environmental science, simulating complex climate systems to aid in weather prediction and climate change impact assessment [4]. Overall, generative AI models like ChatGPT are equipping scientists with advanced tools for processing extensive data, discerning patterns, and uncovering insights that might otherwise be overlooked.

## HARNESSING GENERATIVE AI: EMPOWERING SCIENTIFIC INNOVATION

The advent of generative AI technologies has opened new horizons for the scientific community. Generative AI markedly enhances various critical aspects of scientific research, with a prime focus on improving literature review and text analysis. By swiftly screening and summarizing vast volumes of scientific literature, these models provide researchers with essential background information and emerging trends, thereby saving time and enriching research depth and breadth. Generative AI's contribution to enhancing the quality and efficiency of scientific writing is also noteworthy. Leveraging natural language processing, they aid scientists and editors in drafting, proofreading, and formatting, and are particularly beneficial for nonnative English speakers in crafting precise academic English. These tools efficiently organize and format complex data and theories, streamlining scientific communication for greater accuracy and efficacy.

ChatGPT and generative AI are revolutionizing scientific research by aiding in insight analyses and evaluation. These AI systems excel at analyzing extensive datasets, revealing intricate correlations, patterns, and anomalies that might elude human detection. This is particularly beneficial in areas like genomics, epidemiology, and climate science, which involve large‐scale data analysis.

Generative AI can create code snippets from user descriptions, translating natural language into functional code in various programming languages [5]. This is especially beneficial for novices or those using unfamiliar languages. Moreover, these AI systems excel in identifying and correcting errors in code, quickly pinpointing syntactical and logical issues, and providing explanations, thereby accelerating debugging and serving as an educational resource for developers. Additionally, generative AI can enhance code efficiency and maintainability, suggesting refactoring to improve readability and performance, in line with software development best practices.

Generative AI is enhancing interdisciplinary collaboration and brainstorming by offering suggestions, analogies, and examples from various disciplines. This approach broadens the scope of ideas, encourages thinking beyond conventional boundaries, and fosters creative problem‐solving, which is crucial in fields that thrive on innovation. By providing diverse perspectives and unexpected connections, generative AI can challenge conventional thinking and encourage creative problem‐solving.

ChatGPT and similar AI technologies are significantly improving how scientists communicate with the public, enhancing the spread and comprehension of scientific knowledge. They simplify complex scientific content into more accessible language, overcoming the jargon barrier that often alienates the general public. Correspondingly, an AI virtual persona can be designed for the iMETA journal, tasked with summarizing, condensing, and presenting articles, thus enhancing dissemination and outreach. AI could even analyze articles, substituting for authors in engaging with readers. This demystifies science, making it more approachable. Additionally, in language translation, AI plays a key role in bridging language gaps in scientific communication, ensuring global accessibility, and fostering inclusive scientific discourse.

## MAXIMIZING EFFICIENCY: TIPS FOR UTILIZING GENERATIVE AI

**Figure 1:**
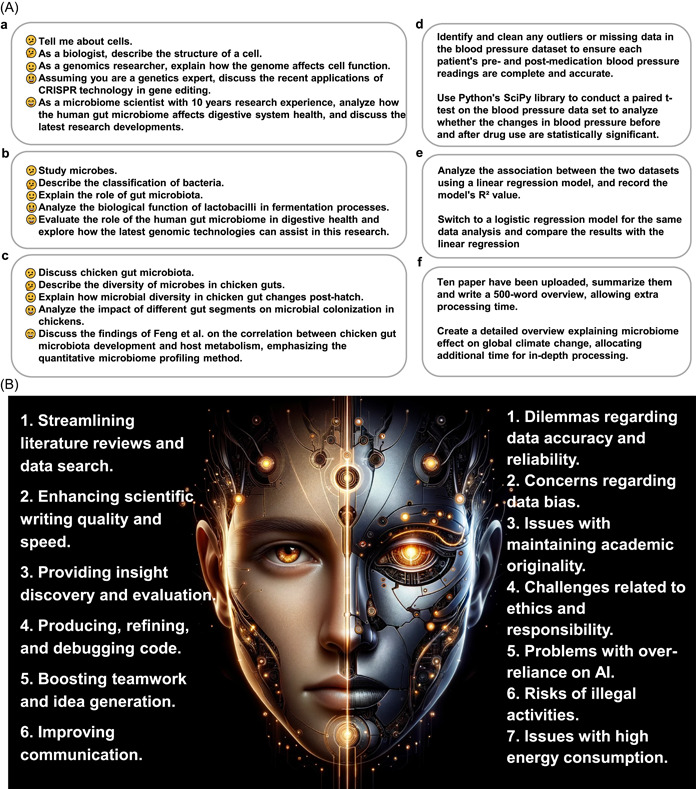
Overview of strategies and the impact of generative AI in scientific research. (A) The various strategies and prompts to enhance the efficiency of generative AI applications, including role‐playing (a), crafting clear instructions (b), providing reference text (c), using external tools (d), systematically testing changes (e), and giving the model time to think (f). (B) Pros and cons of generative AI impact on the scientific community. AI, artificial intelligence.

Prompt engineering is a crucial technique for optimizing generative AI interactions (Figure 1A). This process entails not just instructing the AI on the task at hand, but also formulating requests to maximize efficiency and output quality. Effective prompt engineering involves selecting relevant keywords, providing adequate context, and clearly defining objectives and expectations. Additionally, tailoring prompts to the AI model's specific capabilities and constraints is essential for enhancing performance. Herein, we provide a concise guide on leveraging prompt engineering to boost generative AI efficiency.

Role‐playing occupies a distinct and fascinating niche in prompt engineering. It involves users crafting prompts by emulating various characters or scenarios, thereby drawing out more imaginative and contextually detailed AI responses. For instance, users may prompt the AI to adopt the role of a biochemist, microbiologist, or bioinformatician, or frame inquiries within a specific narrative or context. The specificity and focus of prompts on certain problems or techniques enhance their effectiveness in role‐playing scenarios (Figure 1Aa). The essence of this strategy lies in steering AI toward responses that transcend standard, direct answers, fostering deeper and more nuanced engagement.

In prompt, the clarity of instructions is crucial for eliciting specific, relevant, and high‐quality outputs from generative AI. Well‐defined prompts reduce ambiguity, enhancing AI response efficiency and accuracy. Effective strategies encompass specificity, relevant contextual information, clear parameters, straightforward language, and logical instruction structure to prevent misunderstandings. In microbiology and genomics examples (Figure 1Ab), there is a marked progression from general to highly specific prompts. This shift highlights how detailed instructions improve clarity and guidance, aiding generative AI in more focused and comprehensive research. This methodical approach not only streamlines the research process but also optimizes user experience and conserves resources, making AI interactions more efficient and rewarding.

Incorporating reference texts is crucial for optimizing generative AI, particularly for complex or specialized subjects. This approach enriches the AI's contextual understanding, leading to more precise and relevant outputs. Reference materials also guide the AI in mirroring specific tones and styles, crucial for maintaining consistency with established writing norms or technical vernacular. For example, when summarizing research on the chicken microbiome, utilizing a study from iMETA as a reference (10.1002/imt2.105) significantly improves content quality [6]. A precise instruction might be: “Using the attached paper, write a detailed 500‐word overview about the chicken microbiome.” Such a directive ensures that AI leverages the specific metagenomics context, yielding an informed and accurate summary (Figure 1Ac).

Integrating external tools with generative AI is crucial in enhancing AI‐generated outputs and expanding their capabilities. Tools like data analysis and visualization software complement AI's inherent functions, enabling richer and more intricate outputs. This is particularly beneficial for complex tasks or large datasets, as these tools can process and interpret data beyond AI's standalone capacity. This synergy not only widens the achievable spectrum with AI but also heightens the precision and efficiency of outcomes. For instance, in assessing a new drug's impact on blood pressure, patient data pre‐ and post‐medication could be analyzed. Employing Python's SciPy for a paired *t* test determines the statistical significance of the blood pressure changes. Subsequently, AI, aided by specific prompts, can be used alongside Python to draw definitive conclusions on the drug's effect on blood pressure (Figure 1Ad).

The final two principles essential in AI‐driven research are “systematically testing changes” and “allowing the model adequate processing time.” Systematic variation of parameters and outcome documentation is key to identifying optimal model configurations. This process requires iterative fine‐tuning of AI settings and careful comparison of outcomes to achieve optimal performance. For example, distinct prompts for linear and logistic regression guide the AI in conducting precise statistical analyses in omics research (Figure 1Ae). Conversely, allocating sufficient processing time is vital for complex tasks, permitting the AI to thoroughly handle large datasets or conduct detailed analyses, thus ensuring more accurate and considered results. Specific prompts indicating extended processing time (Figure 1Af), such as “allow extra processing time” or “allocate additional time for in‐depth processing,” instruct the AI to undertake thorough examination and analysis. This approach enhances the depth and quality of outputs across various complex tasks, including research analysis, data interpretation, and intricate problem‐solving.

## MISUSING GENERATIVE AI: RISKS TO SCIENTIFIC INTEGRITY

While generative AI offers considerable advantages to the scientific community, its usage also presents potential drawbacks, encompassing not only the quality and reliability of research but also ethical and responsibility concerns. Diligent usage by scientists and editors is essential to mitigate these issues.

A primary concern is the accuracy and reliability of AI‐generated data. A survey revealed that 66% of scientists are wary of potential errors or inaccuracies infiltrating their research papers [7]. AI models, trained on existing data sets, might propagate outdated, biased, or incomplete information, impacting the output's relevance and accuracy. For the sake of maintaining commercial advantages, the architecture, training data, and scale of most generative AI models are not publicly disclosed. When using data analysis plugins, inputting data can yield analysis results. However, researchers lacking proper data analysis methodologies may struggle to discern the correctness of these methods and determine if errors occurred during the analysis process. It is imperative for scientists to rigorously evaluate AI‐generated content against authoritative sources, a process vital for maintaining accuracy in complex scientific contexts. Generative AI should function as an auxiliary tool augmenting research, rather than replacing human expertise. While beneficial for preliminary data processing, idea generation, or draft creation, the ultimate responsibility for the accuracy and integrity of the final output rests with human experts.

Data bias in ChatGPT and other generative AI models poses a significant challenge, especially in scientific contexts. These AI systems develop their text generation and interpretation capabilities based on their training datasets. Inherently biased data, characterized by stereotypes, prejudices, or skewed representations, can lead to AI perpetuating these biases in its outputs. For example, if training data consistently associates certain concepts or groups, the model may reinforce these biases, thereby deepening existing prejudices. It is crucial for scientists to critically assess generative AI outputs, particularly when they impact research findings or data interpretation. This necessitates vigilance against potential biases and cross‐verification with impartial data sources. Furthermore, educating the scientific community about the propensity for bias in AI models is imperative. Heightened awareness promotes more prudent and informed utilization of AI in research.

The challenge of maintaining academic originality when employing generative AI in scientific research and publication is paramount. While AI excels in processing and synthesizing information, its ability to generate new insights or conduct independent research can be limited by the scope of its training data, though it is increasingly adept at identifying unseen patterns within complex data sets. This raises concerns about originality and plagiarism, with 68% of researchers in a survey indicating that AI could simplify plagiarism and complicate its detection [7]. Although AI‐generated content detectors are being developed to differentiate between AI and human academic writing [8], fostering an ethical academic culture in AI usage is crucial for ensuring proper research conduct. AI should be employed as a supplementary tool in the research process, assisting in drafting, literature review, and ideation. However, the core insights and innovative contributions must stem from the researchers. AI outputs require critical review and adaptation, ensuring that the final work showcases the researchers' unique insights and significantly modifies any AI‐generated content.

The use of generative AI raises critical ethical and responsibility issues due to its transformative impact on academia and society. In scientific writing and research, attributing authorship becomes complicated with AI contributions. While AI cannot be credited as co‐authors in research papers [9], their role should be transparently acknowledged, such as in the acknowledgments section, to preserve the integrity of authorship. When data is frequently retrieved or uploaded to ChatGPT, significant privacy and security concerns emerge, especially when the data involved is sensitive or confidential. This scenario heightens the risk of unauthorized third‐party access and data theft. Furthermore, the training of generative AI on datasets that may include personal or sensitive information exacerbates these privacy issues, presenting additional challenges in safeguarding such data against potential breaches. Inaccurate or unreliable AI outputs also pose a risk of misinformation dissemination. Therefore, the scientific community must establish and follow ethical guidelines and frameworks for using generative AI in research. These should address data usage, bias, transparency, and accountability.

Excessive reliance on ChatGPT may undermine independent thinking and innovation among researchers, particularly in education and training. AI tools, offering rapid access to information, can inadvertently encourage users to accept AI‐generated solutions uncritically. This reliance can diminish personal problem‐solving skills and weaken critical thinking over time. To counter this, researchers and students should be educated to scrutinize and question AI‐provided information, recognizing AI as an aid rather than a definitive source. In educational contexts, AI should complement, not replace, traditional learning methods, ensuring a balanced integration. Essentially, while AI serves as a valuable tool for exploring various solutions, researchers and learners must also cultivate and trust their own problem‐solving skills.

Generative AI misuse poses a multitude of additional risks, including the potential for crafting malware, phishing, and fraud, necessitating robust regulatory frameworks to prevent abuse. Enhancing user education and public awareness about these risks, along with collaboration with government and legal entities, is vital for developing strategies to curb illegal AI use. Additionally, the high energy demands of AI training and operation raise environmental concerns. Addressing this involves developing more efficient algorithms, utilizing renewable energy for computing centers, and promoting awareness of AI's environmental footprint to encourage sustainable practices. Furthermore, AI's impact on employment, particularly in reducing human labor needs, calls for a focus on human‐machine collaboration. Supporting workforce transitions and continuous education is crucial for adapting to technological advancements. Interdisciplinary research is also needed to fully understand AI's labor market impact and inform policy recommendations.

## GUIDING PRINCIPLES: IMPLEMENTING GENERATIVE AI IN IMETA'S PUBLICATION FOR AUTHORS AND REVIEWERS

In light of discussions in our editorial office and insights from other journals [10], we propose guidelines for integrating generative AI in research and scholarly publishing. First, AI‐assisted technologies, including language models and chatbots, cannot be recognized as authors. The utilization of such AI in research or manuscript preparation must be transparently disclosed. Detailed information about the AI tools used, including specific prompts and versions, is required in submissions. Researchers must address ethical concerns and ensure the accuracy and fairness of AI‐generated content, with authors bearing responsibility for their work's integrity. Manuscripts risk rejection for improper AI use, and AI's role in the review process is prohibited. Generally, AI‐generated images and multimedia are not accepted unless specifically allowed. Compliance with data protection and privacy laws is mandatory when processing personal data. Moreover, copyright and intellectual property issues surrounding AI‐generated content must be considered. Human oversight should always prevail in significant scientific decision‐making. Finally, given AI's rapid evolution, our stance on AI‐created multimedia content may adapt in response to changing copyright laws and industry ethical standards.

Janus, the dual‐faced Roman god symbolizing transitions and beginnings, aptly represents the duality of generative AI with LLMs. As 2023 marks the year of their broader application in technology, business, and society, these models bring both benefits and challenges (Figure 1B and Supporting Information: Figure S1). Scientists and editors must recognize these risks and adopt measures to address them. The scientific community could introduce standardized operational guidelines aimed at mitigating risks stemming from the diverse expertise levels among users. This initiative is designed to promote uniformity and reliability in research outcomes. Additionally, a regulatory system plugin for AI usage could be launched, which will continuously monitor researchers' AI interactions. This system is intended to ensure adherence to best practices and will generate a comprehensive supervisory report upon the conclusion of research activities. Effective risk mitigation involves exercising critical judgment and maintaining academic integrity. Researchers should synergize their expertise with AI outputs, rather than relying solely on AI. Additionally, stringent compliance with data protection and privacy laws is vital when handling sensitive data. A balanced approach, combining AI use with independent thinking skill development, enables researchers to leverage these tools while preserving the quality and innovation of scientific research.

Note: The opinion described in this article is based on the author's understanding of the current stage of ChatGPT and generative AI. Our opinion may change depending on how ChatGPT and generative AI develop in the future.

## AI STATEMENT

ChatGPT 4.0 was used for grammar correction and ChatGPT image generator was used to draw Figure 1B.

## AUTHOR CONTRIBUTIONS

Zhongji Pu, Baolei Jia, and Chun‐Lin Shi drafted the paper. All authors have revised, read, and approved the final manuscript for publication.

## CONFLICT OF INTEREST STATEMENT

Baolei Jia is the associate editor of iMeta. Chun‐Lin Shi is the scientific editor of iMeta. Canhui Lan and Yong‐Xin Liu are the Executive Editors of iMeta. Che Ok Jeon is a member of the iMeta editorial board. Shuang‐Jiang Liu and Jingyuan Fu are the Editors‐in‐Chief of iMeta. The authors have declared no competing interests.

## Supporting information


**Figure S1:** The process using ChatGPT image generator to generate the Figure 1B.

## Data Availability

The data that supports the findings of this study are available in the Supporting Information of this article.
