# Supplementary material for: ChatGPT and generative AI are revolutionizing the scientific community: A Janus‐faced conundrum
Source: Imeta. 2024 Feb 27;3(2):e178. doi: 10.1002/imt2.178 (PMC11170961; doi:10.1002/imt2.178)
Supplement: Supplementary file 1 — Figure S1: The process using ChatGPT image generator to generate the Figure 1B. [file IMT2-3-e178-s001.pdf]

image generator

You

Now imagine you are an illustrator

image generator

Great! As an "illustrator," I'm here to help create the image you have in mind. Just describe what you'd like to see, and I'll generate it for you. What illustration can I create for you today?

You

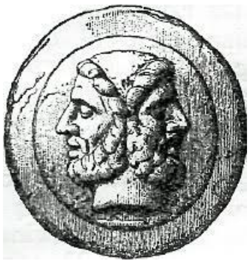

please read the figure. Here's a reminder: He's a two-faced god, male.

image generator

The figure in the image is Janus, the Roman god. He is typically depicted as having two faces, each looking in opposite directions. This design represents duality, such as the transition between past and future, and the concepts of beginnings and endings. Janus is often associated with doorways, gates, and passages, symbolizing his role as a god of transitions.

You

Please use this picture as a template, draw a picture of a face sci-fi in nature, with a ratio of 16 9； the background is black, put the face in the middle, symmetrical on the left and right, so that the left side of face has a human temperament, and the right side has a science fiction， robust and AI temperament. allocating additional time for in-depth processing.

image generator

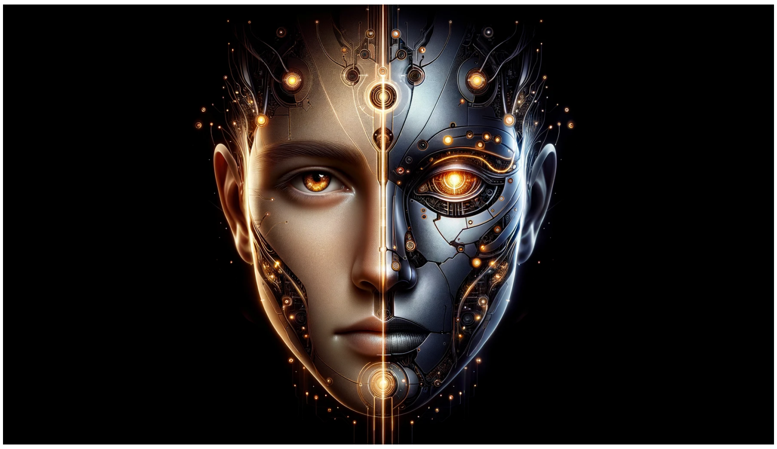

Here is the symmetrical face with a sci-fi theme you requested. The left side exhibits

Message image generator…

ChatGPT can make mistakes. Consider checking important information.

Supporting Information Figure S1. The process using ChatGPT image generator to generate the figure 1B.
